# *De Novo* Assembly of the Whole Transcriptome of the Wild Embryo, Preleptocephalus, Leptocephalus, and Glass Eel of *Anguilla japonica* and Deciphering the Digestive and Absorptive Capacities during Early Development

**DOI:** 10.1371/journal.pone.0139105

**Published:** 2015-09-25

**Authors:** Hsiang-Yi Hsu, Shu-Hwa Chen, Yuh-Ru Cha, Katsumi Tsukamoto, Chung-Yen Lin, Yu-San Han

**Affiliations:** 1 Institute of Fisheries Science, College of Life Science, National Taiwan University, Taipei, Taiwan; 2 Institute of Information Science, Academia Sinica, Taipei, Taiwan; 3 Department of Marine Science and Resources, College of Bioresource Sciences, Nihon University, Fujisawa, Japan; University of Nordland, NORWAY

## Abstract

Natural stocks of Japanese eel (*Anguilla japonica*) have decreased drastically because of overfishing, habitat destruction, and changes in the ocean environment over the past few decades. However, to date, artificial mass production of glass eels is far from reality because of the lack of appropriate feed for the eel larvae. In this study, wild glass eel, leptocephali, preleptocephali, and embryos were collected to conduct RNA-seq. Approximately 279 million reads were generated and assembled into 224,043 transcripts. The transcript levels of genes coding for digestive enzymes and nutrient transporters were investigated to estimate the capacities for nutrient digestion and absorption during early development. The results showed that the transcript levels of protein digestion enzymes were higher than those of carbohydrate and lipid digestion enzymes in the preleptocephali and leptocephali, and the transcript levels of amino acid transporters were also higher than those of glucose and fructose transporters and the cholesterol transporter. In addition, the transcript levels of glucose and fructose transporters were significantly raising in the leptocephali. Moreover, the transcript levels of protein, carbohydrate, and lipid digestion enzymes were balanced in the glass eel, but the transcript levels of amino acid transporters were higher than those of glucose and cholesterol transporters. These findings implied that preleptocephali and leptocephali prefer high-protein food, and the nutritional requirements of monosaccharides and lipids for the eel larvae vary with growth. An online database (http://molas.iis.sinica.edu.tw/jpeel/) that will provide the sequences and the annotated results of assembled transcripts was established for the eel research community.

## Introduction

The Japanese eel, *Anguilla japonica*, is a typical catadromous fish found in the waters of Japan, Korea, China, Taiwan, and the Northern Philippines [1–3]. In East Asia, the Japanese eel is a highly-valued aquaculture species. Eel aquaculture is entirely dependent upon the use of wild glass eels as seedlings because artificial propagation of Japanese eel at the commercial scale is not yet developed. Unfortunately, overfishing, habitat destruction, and changes in the ocean environment have led to the drastic depletion of natural eel stock over the past few decades [4–6]. Therefore, in order to reduce the exploitation of the natural resource, developing the techniques for commercial scale artificial propagation should be a top priority [7].

The artificial propagation of the Japanese eel has been studied for a long time. Yamamoto and Yamauchi [8] first succeeded in producing fertilized eel eggs and larvae using hormone treatments. Many researchers subsequently obtained eel larvae utilizing modified methods of hormone treatments [9–11]. In 1998, Tanaka further indicated that yolk sac and oil droplets of eel larvae would be completely absorbed at 7–9 days post-hatching (dph) [12]. After consuming the yolk sac and oil droplets, feeding becomes very important for the eel larvae.

In previous research, sustained growth and survival were not achieved in artificially produced eel larvae when they were fed with the larvae of mussels, rotifers, or other zooplankton [10, 13, 14]. After prolonged research, Tanaka et al. [15] formulated a slurry-type feed made from shark-egg powder and a soybean peptide, which is appropriate for feeding artificially produced eel larvae. These eel larvae survived for over 200 days and grew to an average total length (TL) of 31 mm. A further modification to the feed formula was made by adding krill hydrolysate and replacing the original soybean peptide with another soybean peptide treated with phytase [16]. Larvae eating this new diet developed into fully grown leptocephali, and then metamorphosed into glass eels, approximately 200–250 dph. Recent studies have also suggested that wild eel larvae may feed on marine snow, fecal pellets of zooplankton, or larvacean houses, which are consistently present on the ocean surface [17–19].

Although the complete culture of the Japanese eel in captivity was achieved in 2010 [20, 21], the growth rate of the artificially produced larvae (0.1–0.3 mm/day) was only half that of the wild larvae (0.5 mm/day) [22, 23]. In addition to growth rate, the survival rate of artificially produced larvae was also very low [7]. Slow growth and low survival rates may occur because the slurry-type feed is not well digested and absorbed in the intestine of eel larvae. In the past, several substantial findings regarding the development of the digestive systems of artificially produced Japanese eel larvae were reported. First, Otake (1996) indicated that the digestive tract of preleptocephali was formed within twenty-four hours after hatching [24]. Next, the pancreas developed around the transitional region between the esophagus and the intestine at 3 dph, and the stomach did not differentiate until the leptocephali metamorphosed into glass eel [25]. Furthermore, the synthesis of pancreatic enzymes (trypsin, chymotrypsin, lipase, and amylase) was initiated within 8 dph [26], and all of them were partially characterized [27]. Additionally, Ozaki et al. (2006) suggested that the absorptive capacity of the alimentary canal was fully developed at 7 dph [28]. However, other digestive enzymes and nutrient transporters of Japanese eel are not well studied, and no study has addressed the food digestion and nutrition absorption during the early developmental stages.

Because of the remarkable advances in computational and sequencing technology such as next-generation sequencing (NGS), the production of genomic-scale data has become simpler [29]. For example, transcriptomic information can be obtained from RNA-seq using next-generation sequencing and provide fundamental insights into biological processes and applications such as levels of gene expression in specific tissues or developmental stages [30], gene expression profiles after experimental treatments [31], gene expression in response to environmental pollution [32, 33], discovery of tissue biomarkers [34], among others. In this study, wild embryos, preleptocephali, leptocephali, and glass eel of the Japanese eel were collected for RNA-seq and transcriptomic assembly. Our goals were to fill in the gaps of the transcriptomic database with the early developmental stages, investigate the transcript levels of genes coding for digestive enzymes and nutrient transporters, and use these transcript levels to evaluate the capacities for nutrient digestion and absorption during larval development. Moreover, because stable growth has not been attained in artificially produced eel larvae fed with only one kind of slurry-type feed, it is vital to clarify whether nutritional requirements vary with different stages in early development for Japanese eel.

## Materials and Methods

### Ethics statement

Embryos, preleptocephali, and leptocephali of the Japanese eel were captured in the Northwest Pacific Ocean (14–15°N, 139–140°E). As this area is in International waters, collection at this location was legal. The glass eels were caught in the estuary of the Yilan River in Taiwan. The Yilan country government and Fisheries Agency of the Council of Agriculture, Executive Yuan, (Taiwan, ROC) issued a permit to us allowing collection activities in this area. Moreover, we also applied for an “Approval of Animal Use Protocol” from the Animal Research Committee of the National Taiwan University, and it was reviewed by the Institutional Animal Care and Use Committee (IACUC). Our IUCAC approval Number is “NTU-101-EL-100”.

### Sample collection

Biopsies of the Japanese eel at four different developmental stages were conducted in this study: unhatched embryos (ten eggs, mean diameter: 1.6 mm, June, 2012), preleptocephali (five individuals, body length: 5.44 ± 0.36 mm, June, 2012), and leptocephali (three individuals, body length: 19.3 ± 5.1 mm, June, 2012) collected in the Northwest Pacific Ocean, and glass eels caught in the estuary of the Yilan River in Taiwan (one on March 14, 2013 and six in April, 2015). These biopsies were preserved in RNA*later* RNA Stabilization Reagent (QIAGEN, Valencia, CA, USA) and stored at -20°C for subsequent total RNA extraction. Unhatched embryos, preleptocephali, leptocephali, and one glass eel were used in the NGS study, and six glass eels were used in the experiments of RT-qPCR.

### RNA extraction, library construction, and sequencing

The total RNA of entire mass of the biopsies was extracted using Trizol® Reagent (Invitrogen, Carlsbad, CA, USA) according to the manufacturer’s instructions. Purified RNA was quantified using a ND-1000 spectrophotometer (Nanodrop, Wilmington, DE, USA) and characterized by a Bioanalyzer 2100 with a RNA 6000 labchip kit (Agilent Technologies, Santa Clara, CA, USA). After analysis with the Bioanalyzer 2100, the numbers of all RNA samples prepared in this study were greater than seven. Sequencing libraries of the unhatched embryos, preleptocephali, leptocephali, and glass eel were constructed using Illumina TruSeq RNA Sample Prep Kits v2 (Illumina, San Diego, CA, USA) according to the manufacturer’s instructions, and they were subsequently sequenced using the Illumina HiSeq 2000.

### Processing of sequence data and *de novo* assembly

Four raw RNA-seq datasets, Fertilized Egg (SRA, NCBI: SRR1930110), Preleptocephalus (SRA, NCBI: SRR1930112), Leptocephalus (SRA, NCBI: SRR1930115) and Glass eel (SRA, NCBI: SRR1930117) were obtained after sequencing. The raw RNA-seq data were filtered using the TrimGalore program (Babraham Bioinformatics, Cambridge, UK), to discard adaptors and low-quality reads (Q < 13). Then, low complexity reads (repeat sequences) were removed using the prinSeq program [35]. Finally, the general read properties were generated using the FastQC program (Babraham Bioinformatics, Cambridge, UK).

The Trinity program (SourceForge, http://trinityrnaseq.sf.net) [36], a commonly used method for the efficient and robust *de novo* reconstruction of transcriptome, was utilized to assemble the transcriptome of the Japanese eel. Following assembly, the counts of transcripts and the N50 were calculated. In addition, open reading frames of the assembled transcripts were predicted using TransDecoder (SourceForge, http://transdecoder.sf.net).

### Functional annotation

Trinotate was used to perform the functional annotations of the transcriptomic data of the Japanese eel. The homologous genes of the protein-coding transcripts were found by comparing the transcripts to the SwissProt database. The transcripts were blasted against the Pfam database [37] to identify specific protein domains and to acquire gene ontology (GO) annotations. The SignalP [38] and tmHMM [39] were used to predict the signal peptide and transmembrane regions of the transcripts. These transcripts were also blasted against the nr database to increase the number of matched homologous genes [40]. Furthermore, these protein-coding transcripts were annotated using the KEGG (Kyoto Encyclopedia of Genes and Genomes) database on the KAAS (KEGG Automatic Annotation Server, http://www.genome.jp/tools/kaas/), setting a parameter for only mapping to the eukaryotic database [41]. The KEGG database contained many important pathways that participate in the regulation of physiological function and development [42, 43]. The single-directional best hit (SBH) method was used to obtain the best match from the KEGG database.

### Gene expression analysis

The assembled transcripts were utilized as templates, and all short reads from the Paired-End data were mapped to the assembled transcripts using the Bowtie program [44]. Subsequently, RSEM (RNA-Seq by Expectation Maximization) [45] was used to calculate the FPKM (Fragments per Kilobase of exon per Million fragments mapped) values of the assembled transcripts [46, 47]. The formula is as follows:

$$FPKM=\frac{mapped\;specific\;exon\;fragments}{total\;mapped\;exon\;fragments\;\left(millions\right)\;*specific\;exon\;length\;\left(KB\right)}$$

In this study, transcript levels of genes coding for digestive enzymes and nutrient transporters specifically existing in the digestive tract were investigated, and all the gene name abbreviations followed the zebrafish nomenclature. Initially, the targeted transcripts of digestive enzymes and nutrient transporters were selected through annotated KEGG pathways. However, each digestive enzyme or transporter may contain many targeted transcripts, some of which were probably alternative splicing forms. Digestive enzymes and nutrient transporters should have no expression at the embryonic stage because the digestive tract was not formed at that time. This was viewed as the standard to select reasonable transcripts of digestive enzymes and nutrient transporters.

### Reverse transcription quantitative PCR (RT-qPCR)

The first strand of cDNA was synthesized from 1 μg of total RNA using HiScript Reverse Transcriptase (Bionova, Fremont, CA, USA) according to the manufacturer’s instructions. The primers of six target genes for RT-qPCR were designed based on the assembled sequences, and listed in Table 1. The acidic ribosomal phosphoprotein P0 (*arp*) which has constant expression in the Japanese eel was used as a reference gene [48]. To determine the specificity of each primer pair, PCR analysis was performed on a cDNA pool. Subsequently, the PCR product was checked for the expected band size (Table 1) via gel electrophoresis, and it was further validated for sequence correctness by DNA sequencing.

**Table 1:** Nucleotide sequence of primers used in real-time quantitative PCR (*arp*: acidic ribosomal phosphoprotein P0, *pep*: pepsinogen, *lip*: triglyceride lipase, *amy*: α-amylase, *slc7a8*: large neutral amino acids transporter small subunit 2, *sglt1*: sodium/glucose co-transporter member 1 and *npc1l1*: Niemann-Pick C1-Like 1).

Gene	Primer	Sequence	Amplicon size (bp)	Efficiency
***arp***	Fw	5’-GTGCCAGCTCAGAACACTG-3’	107	99.8%
	Rv	5’-ACATCGCTCAAGACTTCAATGG-3’		
***pep***	Fw	5’-TCAACCCTCAGGCTTCCTCTA-3’	108	99.6%
	Rv	5’-CGACCTCCACAGTGTCGTAG-3’		
***lip***	Fw	5’-TCGATGGGTGTATCATTATCG-3’	101	99.5%
	Rv	5’-CAAATCCACAGTAGGCTCTCA-3’		
***amy***	Fw	5’-ATGGAAGGACGTCCATAGTTC-3’	77	98.9%
	Rv	5’-TGCTAAGTACCGCTCACATTC-3’		
***slc7a8***	Fw	5’-GATGCTGGTGCACTTCTTCA-3’	112	99.7%
	Rv	5’-CACTGACGGTTGTGTTCCTG-3’		
***sglt1***	Fw	5’-GGTCCTCTTCCACGTCCAT-3’	113	99.9%
	Rv	5’-TCTGTATCGCCTGGTCTGG-3’		
***npc1l1***	Fw	5’-ATGTCACATCAGGGTCTTCAA-3’	95	99.2%
	Rv	5’-ATGCCATGAATCTTGAGATGA-3’		

RT-qPCR was performed on a Bio-Rad MyIQ real-time PCR system (Bio-Rad, Hercules, CA, USA), within a 25-μL mixture containing 12.5 μL of 2X SYBR green supermix (Bionova, Fremont, CA, USA), 1 μL of each primer, 1 μL of cDNA template (ten times dilution) and 9.5 μL DEPC-treated water. The RT-qPCR conditions were: (1) Pre-incubation, 95°C for 10 min; (2) Amplification (40 cycles), 95°C for 30 s, 58°C for 45 s, and 72°C for 45 s. The PCR reaction efficiency (Table 1) for each gene assay was determined using a 2-fold serial dilution of pooled cDNA [49]. Six glass eel samples were used to conduct RT-qPCR experiments, and all of them were repeated three times. The RT-qPCR data were analyzed in two steps. First, the *arp* was used to normalize the data by subtracting its Ct value from the Ct value obtained for each reaction (△Ct). Second, the *pep* and *slc7a8* were individually viewed as control genes for the different categories, digestive enzymes and nutrient transporters, to normalize the data by subtracting their mean △Ct values from the △Ct values of all genes (△△Ct). The normalized mRNA expressions were calculated as 2^^-(△△Ct)^ and presented as the mean ± SD.

### Constructing a transcriptomic database for the Japanese eel

An online transcriptomic database for the Japanese eel containing four transcriptomic datasets (embryo, preleptocephalus, leptocephalus, and glass eel) was constructed using LAMP system architecture (Linux, Apache, MySQL, and PHP). The annotations of all assembled transcripts from nr, GO, Pfam, SignalP, and tmHMM, as well as expressional overview of the KEGG pathway in the Japanese eel were integrated into the database.

### Statistics

Statistical analyses of all the RT-qPCR data (n = 6) were carried out using SAS Statistical software version 9.2 (SAS Institute, Cary, North Carolina, USA). All the RT-qPCR data were tested for the normality and the homogeneity of variance before conducting the statistical analyses. Comparing the differences among the normalized mRNA expressions of different genes was performed using a one-way ANOVA with the Tukey’s HSD multiple comparison tests, and the P value < 0.05 was considered that the difference was significant.

## Results

### Processing of sequencing data and *de novo* assembly

**Fig 1:**
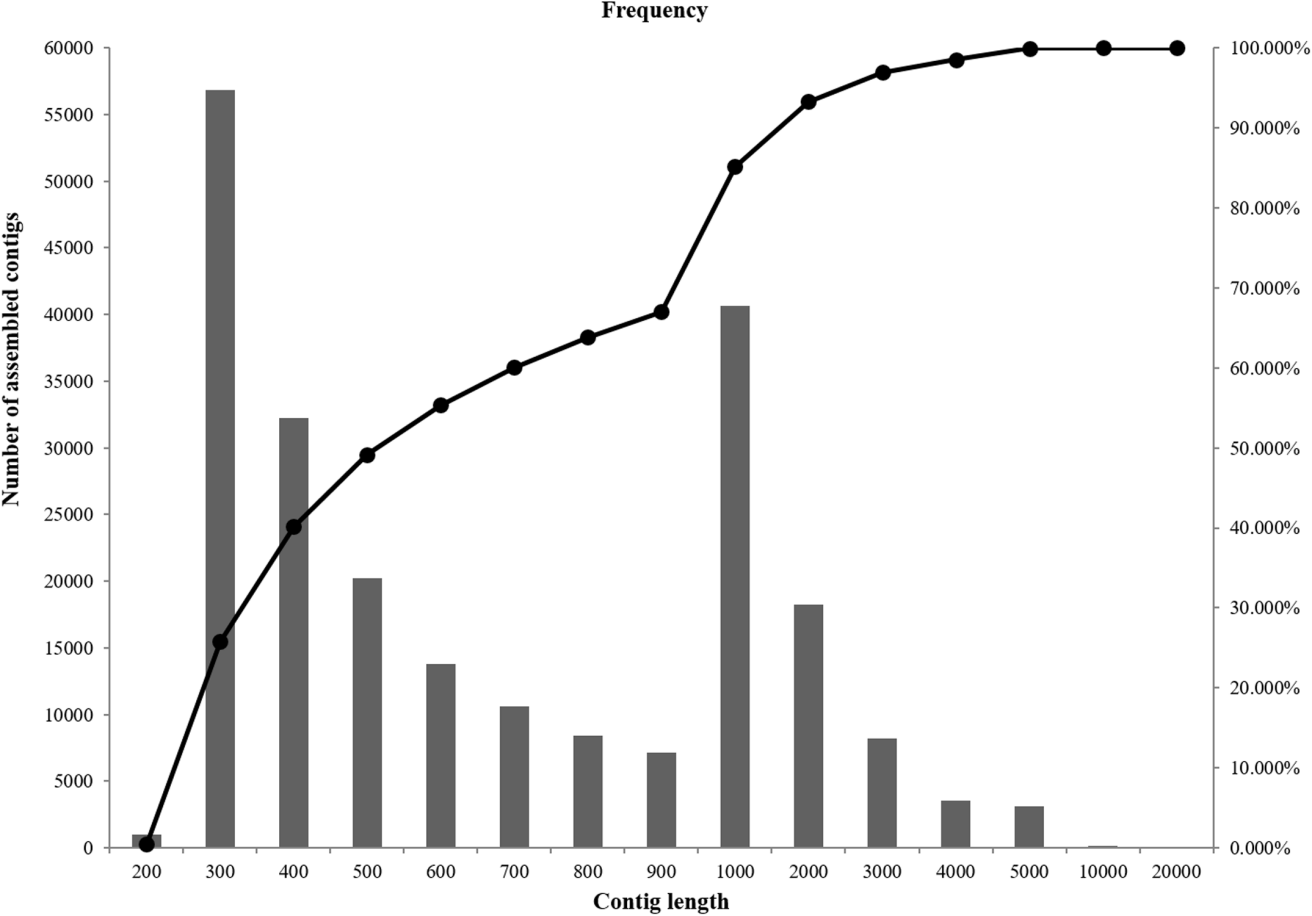
Frequency diagram of contig length This diagram presents an overview of the contigs. In all, approximately 40% of the contigs were <500 bp and 33% of the contigs were >1000 bp. The bar and the solid line individually represent the number of assembled contigs in a specific length interval and the cumulative percentage over a certain length.

The RNA sequencing generated a total of 291,159,035 raw reads, and 278,935,201 (95.8%) clean reads were obtained after removing TruSeq adaptors and low quality reads (Table 2). Thereafter, a total of 224,043 contigs whose length and FPKM values were higher than 200 bps and 0.1 were produced after *de novo* assembly. The contig N50 was 1818 bp long, and the average contig length was 989 bp (Table 3). Furthermore, a frequency diagram of the lengths of assembled contigs was composed (Fig 1). Approximately 50% of contigs were shorter than 500 bp, and 33% of the contigs were longer than 1000 bp.

**Table 2:** Read data before and after processing.

Growth stage of Japanese eel	Number of raw read pairs	Number of cleaned read pairs
**Embryo**	61,717,653	59,042,409 (95.7%)
**Preleptocephali**	61,291,290	58,887,496 (96.1%)
**Leptocephali**	65,860,935	63,721,982 (96.8%)
**Glass eel**	102,289,157	97,283,314 (95.1%)
**Total**	291,159,035	278,935,201 (95.8%)

**Table 3:** Statistics of assembled contigs

**Number of contigs**	224,043
**Total assembled bases, bp**	221,672,216
**Average length of contigs, bp**	989.42
**Length of median contig**	512
**Percent of GC**	47.73
**Contig N10**	4825
**Contig N20**	3564
**Contig N30**	2831
**Contig N40**	2275
**Contig N50**	1818

### Functional annotation for protein-coding transcripts

**Fig 2:**
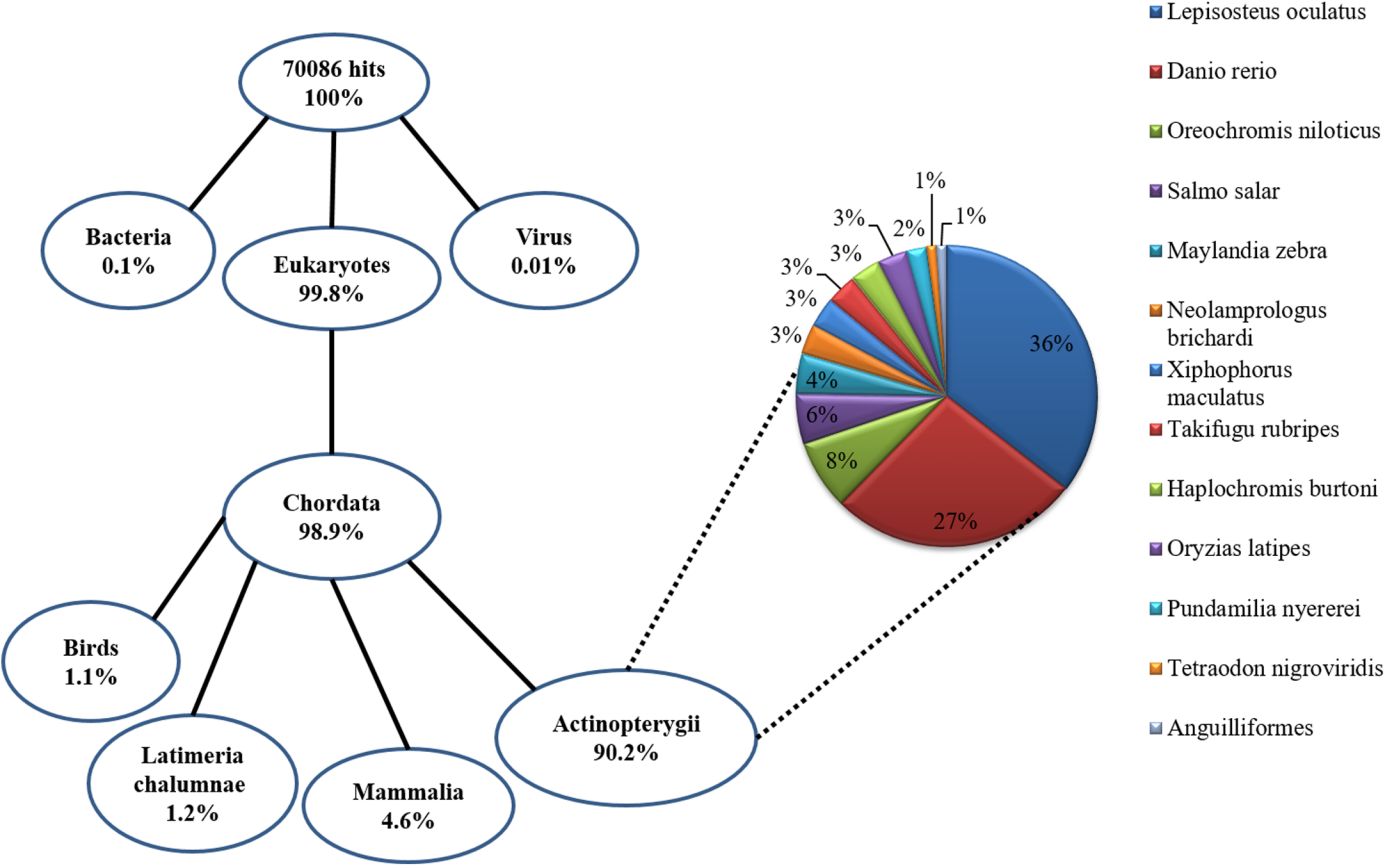
Relative abundance of the main taxonomic groups among the BLAST hits using a simplified Tree of Life diagram Eukaryotes accounted for 99.8% of all positive hits, and Chordata accounted for 98.9%. Among Chordata, Actinopterygii accounted for 90.2% of all positive hits, followed by Mammalia (4.6%). The top-hit species distribution of Actinopterygii was analyzed further: the most heavily represented fish species being *Lepisosteus oculatus* (33%), followed by *Danio rerio* (25%), and *Oreochromis niloticus* (7%).

A total of 116,146 contigs were predicted to be protein-coding transcripts (translated peptides >50 a.a. long). These transcripts were blasted against the SwissProt database (E value < e^-20^), and 21,120 positive matches were found (S1 Table). Pfam, tmHMM, and SignalP were subsequently used to identify specific protein domains, trans-membrane regions, and signal peptides. These transcripts were also blasted against the nr database, and 70,096 corresponding homologous genes were discovered. These homologous genes were submitted for taxonomy analysis, and the results showed that Actinopterygii accounted for 90.2% of all homologous genes, followed by Mammalia (4.6%). The top-hit species distribution of Actinopterygii was further analyzed, and the most representative fish species was *Lepisosteus oculatus* (33%), followed by *Danio rerio* (25%), and *Oreochromis niloticus* (7%) (Fig 2).

### Gene Ontology (GO) analysis

**Fig 3:**
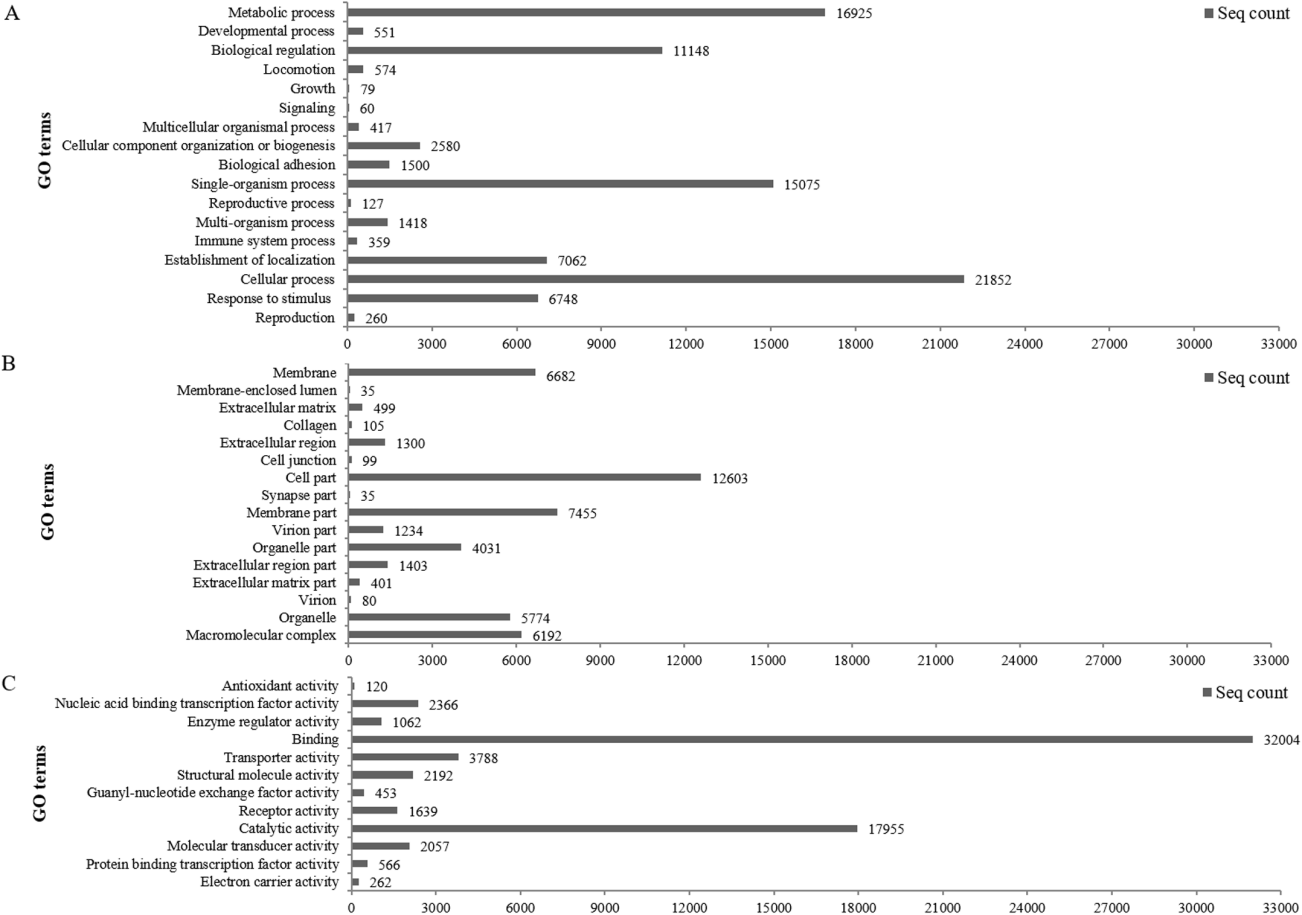
Functional annotation of assembled contigs associated with GO terms (A) In biological processes, 21,852 (25.2%) protein coding transcripts participated in cellular processes, 16,925 (19.5%) transcripts were related to metabolic processes, and 15,075 (17.4%) transcripts were associated with single-organism processes. (B) In the cellular component, 12,603 (26.3%) protein-coding transcripts belonged to cell parts, 7,455 (15.5%) transcripts pertained to membrane parts, and 6,682 (13.9%) protein coding transcripts were part of the membrane. (C) Of those related to molecular functions, 32,004 (49.6%) protein coding transcripts were related to binding, 17,955 (27.8%) were believed to display catalytic activity, and 3,788 (5.9%) transcripts showed transporter activity.

All protein-coding transcripts were classified into three main categories: biological processes, cellular components, and molecular functions. Each category contains many GO terms representing detailed protein functions. In the biological processes category, 21,852 (25.2%) transcripts were related to cellular processes, 16,925 (19.5%) transcripts to metabolic processes, and 15,075 (17.4%) transcripts to single-organism processes (Fig 3A). In the cellular components category, 12,603 (26.3%) transcripts belonged to cell parts, 7,455 (15.5%) transcripts pertained to membrane parts, and 6,682 (13.9%) transcripts were part of the membrane (Fig 3B). In the molecular functions category, 32,004 (49.6%) transcripts were associated with binding, 17,955 (27.8%) transcripts with catalytic activity, and 3,788 (5.9%) transcripts with transporter activity (Fig 3C).

### KEGG pathway analysis

In all protein-coding transcripts, there were 43,033 transcripts matching genes in the KEGG database (37%). Based on the classification by protein family, 21,533 hits were associated with metabolism, 9,424 hits with the processing of genetic information, and 6,956 hits with signaling and cellular processes. A total of 336 KEGG pathways were identified in this study, and the top 10 well-annotated KEGG pathways are listed in Table 4. Although some of these pathways were related to cancer, they may not display the same function in eels. Moreover, some of these pathways assisted in signal transduction regulating the cell’s metabolism, development, and differentiation. The top 10 well-annotated metabolic pathways are shown in Table 5. Nucleic acid metabolism, essential amino acid metabolism, and fatty acid metabolism were found to be important in eel larvae. Inositol phosphates may play crucial roles in cell growth, migration, apoptosis, endocytosis, and differentiation during early development.

**Table 4:** Top 10 well-annotated KEGG pathways.

Rank	Name of pathway	Number of matched KO
**1**	Metabolic pathways	805
**2**	Pathways in cancer	228
**3**	PI3K-Akt signaling pathway	203
**4**	Biosynthesis of secondary metabolites	195
**5**	Neuroactive ligand-receptor interaction	181
**6**	HTLV-1 infection	178
**7**	MAPK signaling pathway	174
**8**	Proteoglycans in cancer	146
**9**	Ras signaling pathway	144
**10**	Focal adhesion	137

KO: KEGG orthology.

**Table 5:** Top 10 well-annotated metabolic pathways.

Rank	Name of pathway	Number of matched KO
**1**	Purine metabolism	115
**2**	Pyrimidine metabolism	73
**3**	Glycerophospholipid metabolism	48
**4**	Protein digestion and absorption	47
**5**	Arginine and proline metabolism	39
**6**	Lysine degradation	39
**7**	Valine, leucine and isoleucine degradation	37
**8**	Amino sugar and nucleotide sugar metabolism	37
**9**	Fatty acid metabolism	35
**10**	Inositol phosphate metabolism	35

KO: KEGG orthology

### Expressional profiles of digestive enzymes at different ontogenetic stages

**Fig 4:**
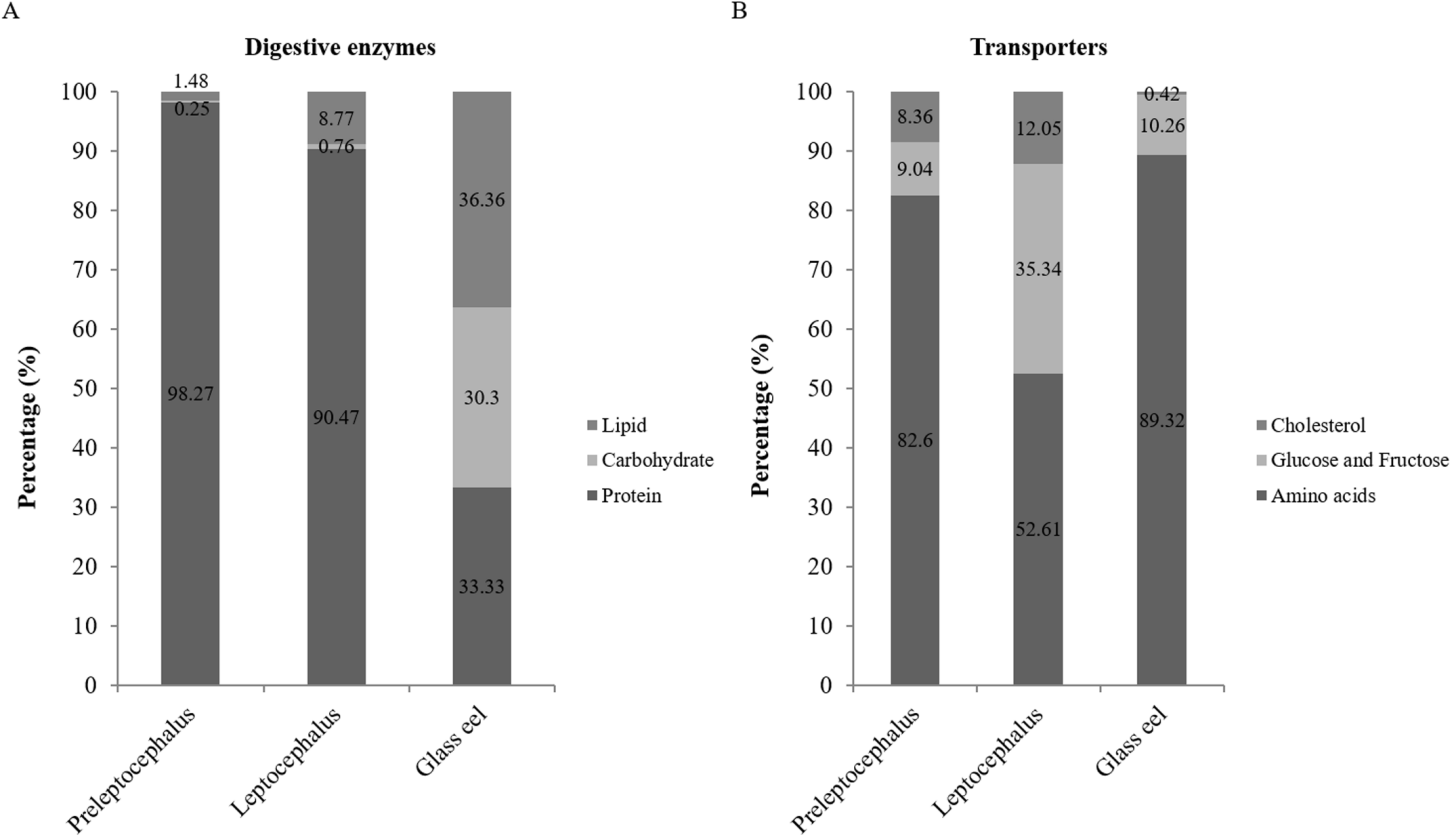
Expressional percentages of different categories of digestive enzymes and nutrient transporters at different stages (A) In preleptocephali, transcript levels of protein digestion enzymes accounted for 98.27% of the total, followed by lipid digestion enzymes (1.48%) and carbohydrate digestion enzymes (0.25%). In leptocephali, the transcript levels of protein digestion enzymes accounted for 90.47% of the total, followed by lipid digestion enzymes (8.77%) and carbohydrate digestion enzymes (0.76%). In glass eels, transcript levels of lipid digestion enzymes accounted for 36.36%, followed by protein digestion enzymes (33.33%) and carbohydrate digestion enzymes (30.3%). (B) In preleptocephali, transcript levels of amino acid transporters accounted for 82.6%, followed by glucose and fructose transporters (9.04%) and cholesterol transporters (8.36%). In leptocephali, transcript levels of amino acid transporters accounted for 52.61%, followed by glucose and fructose transporters (35.34%) and cholesterol transporters (12.05%). In glass eels, transcript levels of amino acid transporters accounted for 89.32%, followed by glucose and fructose transporters (10.26%) and cholesterol transporters (0.42%).

The targeted transcripts of digestive enzymes were selected from the assembled transcripts annotated by the KEGG database (S2 Table). After screening, the targeted transcripts of each digestive enzyme selected for expressional analysis were summarized in S3 Table. The transcript levels of genes coding for digestive enzymes are presented according to the developmental stages in which they are produced (S1 Fig). These enzymes were classified into three categories (protein, carbohydrate, and lipid), and the expressional percentage of each category at different stages was calculated (Fig 4A). The results showed that the transcript levels of protein digestion enzymes were very high in the stages of preleptocephali and leptocephali, but those of lipid and carbohydrate digestion enzymes were low. In addition, the transcript levels of protein, carbohydrate, and lipid digestion enzymes were almost the same in the stage of glass eel.

**Fig 5:**
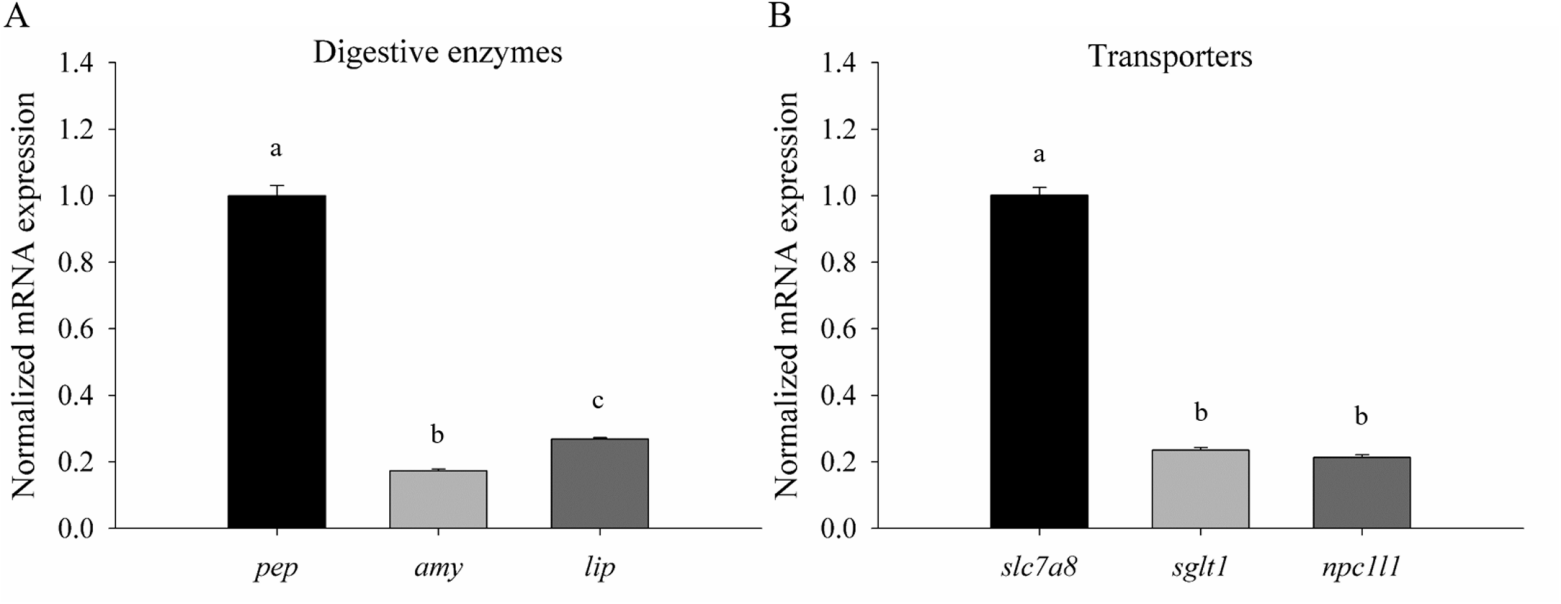
Relative mRNA expression levels of digestive enzymes and nutrient transporters in glass eel The mRNA expression levels were presented as mean ± SD (n = 6). (A) For the digestive enzymes, the mRNA expression level of pepsinogen (*pep*) was approximately six times higher than that of α-amylase (*amy*), and four times higher than that of triglyceride lipase (*lip*). (B) For the nutrient transporters, the mRNA expression level of large neutral amino acids transporter small subunit 2 (*slc7a8*) was approximately four point five times higher than that of sodium/glucose co-transporter member 1 (*sglt1*), and five times higher than that of Niemann-Pick C1-like 1 (*npc1l1*). Means with the same letter were not significantly different at the 5% level, as determined by Tukey’s HSD test.

Next, three representative genes, *pep*, *amy*, and *lip*, were selected for the validation of the transcriptomic results via RT-qPCR. Because samples of wild embryos, preleptocephali, and leptocephali were difficult to collect, only the glass eel can be used for the validation. The RT-qPCR data showed that the gene expression of pepsinogen was approximately six times higher than that of α-amylase, and four times higher than that of triglyceride lipase (Fig 5A), which were consistent with the transcriptomic data.

### Expressional profiles of nutrient transporters existing in intestinal epithelial cells at different ontogenetic stages

The targeted transcripts of nutrient transporters are listed in S4 Table. After screening, the transcripts for expressional analysis were summarized in S5 Table, and the transcript levels of genes coding for nutrient transporters were displayed based on different developmental stages (S2 Fig). Similarly, these nutrient transporters were also classified into three categories (amino acid, glucose and fructose, and cholesterol), and the expressional percentage of each category at different stages was calculated (Fig 4B). The transcript levels of amino acid transporters were the highest in the stages of preleptocephali, leptocephali, and glass eel, followed by glucose and fructose transporters and cholesterol transporter. Moreover, expressional percentage of glucose and fructose transporters was much higher in leptocephali than in preleptocephali and glass eel.

For the nutrient transporters, *slc7a8*, *sglt1*, and *npc1l1* were chosen for validating the transcriptomic results via RT-qPCR. The results of RT-qPCR showed that gene expression of *slc7a8* was approximately four point five times higher than that of *sglt1*, and five times higher than that of *npc1l1* (Fig 5B). However, the transcriptomic results indicated that gene expression of *slc7a8* was nearly twenty-five times higher than that of *sglt1*, and more than one hundred times higher than that of *npc1l1* (S2 Fig). Although the expressional differences among the three genes from the RT-qPCR data were not as great as those from the transcriptomic data, the similar tendency that gene expression of *slc7a8* was higher than those of *sglt1* and *npc1l1* could be observed.

### Online transcriptome database for Japanese eel

An online transcriptomic database for Japanese eel was established in this study, and the URL of the online database was http://molas.iis.sinica.edu.tw/jpeel/. This database integrated the annotations from the BLAST/nr, GO, Pfam, SignalP, tmHMM, and KEGG databases. Users can find the genes that they interested in by entering keywords through “Full-text search on Annotation tables”. Uploading sequences to the “Sequence Search/BLAST” section can blast against the transcriptomic database for discovering homologous genes. Furthermore, users can also search for specific KEGG pathways as well as the annotated results via “KEGG GlobalView”.

## Discussion

Recently, next-generation sequencing has been extensively utilized to reconstruct the genomes [50, 51] and the transcriptomes of aquatic organisms [52–54], which can efficiently improve the quality of basic studies. The first transcriptome research on the Japanese eel was performed on the adult eels, in order to investigate the osmoregulation during adaption to changes in salinity [31]. The gills of silver eels undergoing adaptation to altered salinity were collected for deep sequencing, and a total of 20,826 contigs were generated. All contigs were submitted for taxonomy analysis, in which 11,033 (53%) positive hits were obtained. In our research, the biopsies of early developmental stages of Japanese eel were collected to perform RNA-seq to decipher the digestive and absorptive capacities during larval development.

Detailed knowledge of digestive physiology during larval development in fish species with good potential for aquaculture is essential for the production of healthy fingerlings. In the past, many studies focused on which feed and feed additives were suitable for Japanese eel larvae [14–16], however, the digestive and absorptive capacities were seldom the focus of research. In this study, the results showed that the transcript levels of protein digestion enzymes and amino acid transporters were very high in the stages of preleptocephali and leptocephali. Wild leptocephali mainly fed on marine snow, which is made up of a variety of types of organic matter, including dead or dying gelatinous zooplankton, protists, and fecal matter [17, 18]. These foods are primarily protein detritus, thus, the capacity for protein digestion and amino acid absorption is most important for preleptocephali and leptocephali. In our research, we also found that the transcript levels of carbohydrate-digestion enzymes were low in the stages of preleptocephali and leptocephali, whereas those of glucose and fructose transporters were much higher, indicating that the digestion of carbohydrates would be poor, but the absorption of monosaccharides would be good. A previous study suggested that the gut microbiota of fish may play an important role in nutrient digestion and immunological processes [55]. In addition, activity of β-glucosidase was detected in the bacteria isolated from marine snow [56]. Therefore, the monosaccharides required for the synthesis of hyaluronan, which is the main component of the bodies of preleptocephali and leptocephali [57], may be produced with the help of gut microbiota of eel larvae or microbiota in the marine snow.

Dietary lipids were usually composed of cholesterol and triacylglyceride. Cholesterol can travel through the plasma membrane via transporters, or directly diffuse into the intestinal enterocytes with the help of bile salt [58]. Triacylglyceride must be broken down into free fatty acids and monoglycerides by triacylglyceride lipase and bile salt-stimulated lipase [59], then the free fatty acids and monoglycerides are able to diffuse into intestinal enterocytes with the help of bile salt, or be carried by transporters [60]. In the preleptocephali and leptocephali, the transcript levels of lipid digestion enzymes were higher than those of carbohydrate digestion enzymes. Thus, it appears that the capacity for lipid digestion was better than that of carbohydrate digestion in preleptocephali and leptocephali. In addition, the cholesterol transporter was also expressed in the intestine of preleptocephali and leptocephali, indicating that cholesterol may be absorbed into the body for utilization.

In the stage of glass eel, our results showed that the transcript levels of protein digestion enzymes did not significantly differ from the lipid and carbohydrate digestion enzymes. Moreover, the transcript levels of amino acid transporters were higher than those of glucose and fructose transporters and the cholesterol transporter. The glass eel, which is formed from the metamorphosis of leptocephalus [2], has a well-developed digestive tract and stomach [25]. Therefore, it would have a more balanced performance of enzymes activity than that of the leptocephali. Additionally, in the life cycle of Japanese eel, the stage of glass eel is the main stage during which the Japanese eel begin to enter the rivers that are their main habitat [2]. To enter the rivers, glass eel would need strong muscles and sufficient energy, leading to higher absorptive ability of amino acids.

In this study, the glass eels were used to conduct the RT-qPCR experiments for the validation of transcriptomic data. For the digestive enzymes, the RT-qPCR data were consistent with the transcriptomic data. However, for the nutrient transporters, gene expressions of *slc7a8*, *sglt1* and *npc1l1* were not in line with the transcriptomic data. The main possible reason bringing about the discrepancy between the RT-qPCR data and the transcriptomic data may be individual variance among different individuals. Secondly, according to the formula of FPKM calculation, the length of transcripts and incomplete paired-end data could affect the FPKM value. Although there was discrepancy between the RT-qPCR data and the transcriptomic data, the general tendency was similar in both data.

The nutritional requirements of most marine fish larvae are not well known, and they may differ from those of juveniles and adults owing to the strong morphological and physiological changes during ontogenesis [61]. The eel larvae, preleptocephali and leptocephali, are an extraordinary type of fish larvae because of their extreme lateral body compression and transparency [62]. They also undergo substantial morphological and physiological changes during early development. Therefore, the nutritional requirements of Japanese eel larvae may vary with different stages. A previous study showed that the growth rate of the artificially produced leptocephali, which fed on the *Squalus acanthias* egg-based diet, was lower than that of wild leptocephali [22]. The organic compounds present in the *S*. *acanthias* egg-based diet are protein (26.3%), lipids (17.5%), carbohydrates (0.1%), and moisture (54.4%) [23]. Based on our results, we think that the content of carbohydrates in the *S*. *acanthias* egg-based diet does not satisfy the nutritional requirements of preleptocephali and leptocephali, especially leptocephali. Okamura et al. [23] indicated that the growth rate of early-stage larvae was drastically improved by supplying a diet containing more sugars such as *N-*acetylglucosamine, glucose, and maltose. On the other hand, we also think that the content of lipids in the *S*. *acanthias* egg-based diet would exceed the nutritional requirements of preleptocephali and leptocephali. Furuita et al. [63] found that decreasing dietary lipids can enhance the nutritional value of shark eggs and subsequently increase the survival rate of eel larvae.

These results of previous studies are in agreement with the results of our experiments. Moreover, we further conclude that the nutritional requirements of Japanese eel vary with different stages during early development according to our results. This information will be important as reference for developing adequate feeds and feeding protocols during early development of Japanese eel. For example, at the start of feeding, a high-protein, low-carbohydrate, and low-fat diet could be prepared for the preleptocephali. In addition, the contents of carbohydrates and lipids could be gradually increased as the eel larvae grew. However, determining the actual dietary formulas will require more research.

## Conclusions

To our knowledge, this is the first study focusing on the evaluation of the digestive and absorptive capacities of the Japanese eel during early development. An online transcriptomic database containing many transcripts and detailed annotations has also been established. Our research indicated that the capacity for protein digestion and amino acid absorption may be most important in eel larvae. The capacity for carbohydrate and lipid digestion may be poor in the preleptocephali stage. The nutritional requirements of monosaccharides and lipids appeared to increase as larvae grow. Moreover, at the glass eel stage, the absorption of amino acids may arise from the need to produce strong muscles and sufficient energy to migrate to the rivers. In the future, more detailed information, such as what kinds of amino acids, fatty acids, vitamins, and microelements can be greatly utilized by eel larvae, shall need to be further clarified.

## Supporting Information

S1 Fig Expressional profiles of digestive enzymes categorized by developmental stage. This figure shows the transcript levels of digestive enzymes, which existed in digestive tract at different stages. (A) In the preleptocephalus stage, *ctr*, *cpb*, and *cela3b* were highly transcribed. However, the rest of the enzymes had low or almost nonexistent transcript levels. (B) The transcript levels of the digestive enzymes in leptocephali were similar to those in preleptocephali. (C) In the glass eel stage, *pep*, *chia*.*3*, *mgam*, and *lipf* were more highly transcribed than the rest of enzymes. The gene name abbreviations are as follows; *pep*: pepsinogen, *try*: trypsinogen, *ctr*: chymotrypsin, *cela3b*: chymotrypsin-like elastase family member 3B-like, *cpa2*: carboxypeptidase A2, *cpb*: carboxypeptidase B, *tmprss7*: enteropeptidase (transmembrane protease, serine 7), *chia*.*3*: chitinase, acidic.3, *amy*: α-amylase, *mgam*: maltase-glucoamylase, intestinal-like, *lip*: triglyceride lipase (pancreatic lipase-related protein 1), *clps*: colipase, *bal1*: bile salt-activated lipase 1, and *lipf*: lysosomal acid lipase/cholesteryl ester hydrolase.
(DOCX)

S2 Fig Expressional profiles of nutrient transporters categorized by developmental stage. This figure shows the transcript levels of the nutrient transporters existing in the digestive tract at different stages. (A) In the preleptocephalus stage, *pept1*, *slc31*, and *slc7a8* had higher transcript levels than others. (B) With the exception of *slc2a5*, the rest of transporters had high transcript levels in the leptocephalus stage. (C) In the glass eel stage, *pept1*, *slc7a8*, *sglt1*, and *slc2a2* had higher transcript levels. The gene name abbreviations are as follows; *pept1*: peptide transporter 1, *slc31*: Neutral and basic amino acid transport protein rBAT (solute carrier family 3 (amino acid transporter), member 1), *slc7a8*: Large neutral amino acid transporter small subunit 2 (solute carrier family 7 (L-type amino acid transporter)), member 8), *sglt1*: Sodium/glucose co-transporter member 1, *slc2a5*: solute carrier family 2 (facilitated glucose/fructose transporter) member 5-like, *slc2a2*: solute carrier family 2 facilitated glucose transporter member 2, and *npc1l1*: Niemann-Pick C1-Like protein 1.
(DOCX)

S1 Table Statistical number of assembled sequences BLAST compared against known sequences in SwissProt at different coverage (%). (DOCX)

S2 Table Partial annotation of all targeted transcripts of digestive enzymes specifically existing in the digestive tract. (DOCX)

S3 Table Targeted transcripts of all digestive enzymes for expressional analysis and their FPKM values at different stages. (DOCX)

S4 Table Partial annotation of all targeted transcripts of nutrient transporters existing in the digestive tract. (DOCX)

S5 Table Targeted transcripts of each nutrient transporter for expressional analysis and its FPKM value at different stages. (DOCX)
