# Supplementary material for: De Novo Assembly of the Whole Transcriptome of the Wild Embryo, Preleptocephalus, Leptocephalus, and Glass Eel of Anguilla japonica and Deciphering the Digestive and Absorptive Capacities during Early Development
Source: PLoS One. 2015 Sep 25;10(9):e0139105. doi: 10.1371/journal.pone.0139105 (PMC4583181; doi:10.1371/journal.pone.0139105)
Supplement: S1 Fig — This figure shows the transcript levels of digestive enzymes, which existed in digestive tract at different stages. (A) In the preleptocephalus stage, ctr, cpb, and cela3b were highly transcribed. However, the rest of the enzymes had low or almost nonexistent transcript levels. (B) The transcript levels of the digestive enzymes in leptocephali were similar to those in preleptocephali. (C) In the glass eel stage, pep, chia.3, mgam, and lipf were more highly transcribed than the rest of enzymes. The gene name abbreviations are as follows; pep: pepsinogen, try: trypsinogen, ctr: chymotrypsin, cela3b: chymotrypsin-like elastase family member 3B-like, cpa2: carboxypeptidase A2, cpb: carboxypeptidase B, tmprss7: enteropeptidase (transmembrane protease, serine 7), chia.3: chitinase, acidic.3, amy: α-amylase, mgam: maltase-glucoamylase, intestinal-like, lip: triglyceride lipase (pancreatic lipase-related protein 1), clps: colipase, bal1: bile salt-activated lipase 1, and lipf: lysosomal acid lipase/cholesteryl ester hydrolase. (DOCX) [file pone.0139105.s001.docx]

A





B





C





**S1 Fig. Expressional profiles of digestive enzymes categorized by developmental stage.**

This figure shows the transcript levels of digestive enzymes, which existed in digestive tract at different stages. (A) In the preleptocephalus stage, *ctr*, *cpb*, and *cela3b* were highly transcribed. However, the rest of the enzymes had low or almost nonexistent transcript levels. (B) The transcript levels of the digestive enzymes in leptocephali were similar to those in preleptocephali. (C) In the glass eel stage, *pep*, *chia.3*, *mgam*, and *lipf* were more highly transcribed than the rest of enzymes. The gene name abbreviations are as follows; *pep*: pepsinogen, *try*: trypsinogen, *ctr*: chymotrypsin, *cela3b*: chymotrypsin-like elastase family member 3B-like, *cpa2*: carboxypeptidase A2, *cpb*: carboxypeptidase B, *tmprss7*: enteropeptidase (transmembrane protease, serine 7), *chia.3*: chitinase, acidic.3, *amy*: α-amylase, *mgam*: maltase-glucoamylase, intestinal-like, *lip*: triglyceride lipase (pancreatic lipase-related protein 1), *clps*: colipase, *bal1*: bile salt-activated lipase 1, and *lipf*: lysosomal acid lipase/cholesteryl ester hydrolase.
