# Supplementary material for: De Novo Assembly of the Whole Transcriptome of the Wild Embryo, Preleptocephalus, Leptocephalus, and Glass Eel of Anguilla japonica and Deciphering the Digestive and Absorptive Capacities during Early Development
Source: PLoS One. 2015 Sep 25;10(9):e0139105. doi: 10.1371/journal.pone.0139105 (PMC4583181; doi:10.1371/journal.pone.0139105)
Supplement: S2 Fig — This figure shows the transcript levels of the nutrient transporters existing in the digestive tract at different stages. (A) In the preleptocephalus stage, pept1, slc31, and slc7a8 had higher transcript levels than others. (B) With the exception of slc2a5, the rest of transporters had high transcript levels in the leptocephalus stage. (C) In the glass eel stage, pept1, slc7a8, sglt1, and slc2a2 had higher transcript levels. The gene name abbreviations are as follows; pept1: peptide transporter 1, slc31: Neutral and basic amino acid transport protein rBAT (solute carrier family 3 (amino acid transporter), member 1), slc7a8: Large neutral amino acid transporter small subunit 2 (solute carrier family 7 (L-type amino acid transporter)), member 8), sglt1: Sodium/glucose co-transporter member 1, slc2a5: solute carrier family 2 (facilitated glucose/fructose transporter) member 5-like, slc2a2: solute carrier family 2 facilitated glucose transporter member 2, and npc1l1: Niemann-Pick C1-Like protein 1. (DOCX) [file pone.0139105.s002.docx]

A





B





C





**S2 Fig. Expressional profiles of nutrient transporters categorized by developmental stage.** This figure shows the transcript levels of the nutrient transporters existing in the digestive tract at different stages. (A) In the preleptocephalus stage, *pept1*, *slc31*, and *slc7a8* had higher transcript levels than others. (B) With the exception of *slc2a5*, the rest of transporters had high transcript levels in the leptocephalus stage. (C) In the glass eel stage, *pept1*, *slc7a8*, *sglt1*, and *slc2a2* had higher transcript levels. The gene name abbreviations are as follows; *pept1*: peptide transporter 1, *slc31*: Neutral and basic amino acid transport protein rBAT (solute carrier family 3 (amino acid transporter), member 1), *slc7a8*: Large neutral amino acid transporter small subunit 2 (solute carrier family 7 (L-type amino acid transporter)), member 8), *sglt1*: Sodium/glucose co-transporter member 1, *slc2a5*: solute carrier family 2 (facilitated glucose/fructose transporter) member 5-like, *slc2a2*: solute carrier family 2 facilitated glucose transporter member 2, and *npc1l1*: Niemann-Pick C1-Like protein 1.
