# Supplementary material for: De Novo Assembly of the Whole Transcriptome of the Wild Embryo, Preleptocephalus, Leptocephalus, and Glass Eel of Anguilla japonica and Deciphering the Digestive and Absorptive Capacities during Early Development
Source: PLoS One. 2015 Sep 25;10(9):e0139105. doi: 10.1371/journal.pone.0139105 (PMC4583181; doi:10.1371/journal.pone.0139105)
Supplement: S1 Table — (DOCX) [file pone.0139105.s003.docx]

**S1 Table. Statistical number of assembled sequences BLAST compared against known sequences in SwissProt at different coverage (%)**

| Coverage (%) | Count | Cumulative counts |
| --- | --- | --- |
| 100 | 8021 | 8021 |
| 90 | 1834 | 9855 |
| 80 | 1376 | 11231 |
| 70 | 1375 | 12606 |
| 60 | 1565 | 14171 |
| 50 | 1762 | 15933 |
| 40 | 1738 | 17671 |
| 30 | 1834 | 19505 |
| 20 | 1615 | 21120 |
| 10 | 0 | 21120 |
| 0 | 0 | 21120 |
