# Supplementary material for: De Novo Assembly of the Whole Transcriptome of the Wild Embryo, Preleptocephalus, Leptocephalus, and Glass Eel of Anguilla japonica and Deciphering the Digestive and Absorptive Capacities during Early Development
Source: PLoS One. 2015 Sep 25;10(9):e0139105. doi: 10.1371/journal.pone.0139105 (PMC4583181; doi:10.1371/journal.pone.0139105)
Supplement: S2 Table — (DOCX) [file pone.0139105.s004.docx]

**S2 Table. Partial annotation of all targeted transcripts of digestive enzymes specifically existing in the digestive tract**

| Contig ID | Contig length(bp) | Protein length(a.a.) | KEGG ID & name | (nr) Hit_organism | (nr) Hit_annotation | (nr) Identity |
| --- | --- | --- | --- | --- | --- | --- |
| comp151497_c0_seq1 | 700 | 232 | K06002 (Pepsin A) | *Anguilla japonica* | pepsinogen | 99.57% |
| comp167887_c0_seq1 | 523 | 134 | K06002 (Pepsin A) | *Anguilla japonica* | pepsinogen | 100% |
| comp323922_c0_seq1 | 266 | 88 | K06002 (Pepsin A) | *Lepisosteus oculatus* | pepsin A-like | 74.71% |
| comp272674_c0_seq1 | 218 | 72 | K06002 (Pepsin A) | *Lepisosteus oculatus* | pepsin A-like | 78.08% |
| comp161989_c0_seq1 | 1330 | 402 | K06002 (Pepsin A) | *Homo sapiens* | pepsin A preprotein | 100% |
| comp141995_c0_seq2 | 420 | 60 | K01312 (Trypsin) | *Xiphophorus maculatus* | trypsin I-P1-like | 84.62% |
| comp143629_c0_seq1 | 830 | 67 | K01312 (Trypsin) | *Xiphophorus maculatus* | trypsin I-P1-like | 78.46% |
| comp150796_c0_seq1 | 806 | 73 | K01312 (Trypsin) | *Lepisosteus oculatus* | trypsin-3-like | 84.72% |
| comp165548_c0_seq1 | 660 | 155 | K01312 (Trypsin) | *Acartia pacifica* | trypsin | 71.9% |
| comp165548_c0_seq2 | 892 | 263 | K01312 (Trypsin) | *Acartia pacifica* | trypsin | 65.59% |
| comp172237_c0_seq1 | 707 | 235 | K01312 (Trypsin) | *Drosophila melanogaster* | Beta Trypsin | 99.15% |
| comp172237_c0_seq2 | 623 | 207 | K01312 (Trypsin) | *Drosophila melanogaster* | alphaTrypsin | 98.07% |
| comp189910_c0_seq1 | 2875 | 248 | K01312 (Trypsin) | *Salmo salar* | Trypsin precursor | 77.02% |
| comp184260_c0_seq1 | 878 | 254 | K01312 (Trypsin) | *Osmerus mordax* | Anionic trypsin-1 precursor | 82.11% |
| comp175835_c1_seq1 | 664 | 221 | K01312 (Trypsin) | *Solea senegalensis* | trypsinogen 2 | 78.61% |
| comp175835_c1_seq3 | 432 | 93 | K01312 (Trypsin) | *Anguilla japonica* | trypsinogen | 96.77% |
| comp175835_c1_seq2 | 608 | 202 | K01312 (Trypsin) | *Anguilla japonica* | trypsinogen | 76.4% |
| comp197696_c0_seq1 | 1176 | 247 | K01312 (Trypsin) | *Epinephelus coioides* | trypsinogens 1 | 83.33% |
| comp197696_c0_seq2 | 855 | 247 | K01312 (Trypsin) | *Paralichthys olivaceus* | trypsinogen 2 precursor | 83.33% |
| comp167629_c0_seq1 | 1424 | 159 | K01312 (Trypsin) | *no* | no | no |
| comp191757_c0_seq1 | 1113 | 264 | K01310 (chymotrypsin) | *Thunnus orientalis* | chymotrypsinogen 2 | 86.31% |
| comp195660_c0_seq7 | 1377 | 264 | K01310 (chymotrypsin) | *Thunnus orientalis* | chymotrypsinogen 1 | 78.79% |
| comp189655_c1_seq1 | 1062 | 275 | K01346 (pancreatic elastase II) | *Sparus aurata* | unknown | 67.66% |
| comp176496_c0_seq1 | 768 | 230 | K01346 (pancreatic elastase II) | *Paralichthys olivaceus* | elastase 3 precursor | 81.22% |
| comp177953_c0_seq1 | 1018 | 304 | K01345 (pancreatic endopeptidase E) | *Xiphophorus maculatus* | chymotrypsin-like elastase family member 3B-like | 65.06% |
| comp214687_c0_seq1 | 496 | 162 | K01346 (pancreatic elastase II) | *Danio rerio* | novel elastase protein | 99.38% |
| comp194055_c0_seq1 | 1314 | 377 | K08779 (carboxypeptidase A1) | *Salmo salar* | Carboxypeptidase A1 precursor | 77.93% |
| comp192750_c0_seq1 | 1797 | 430 | K01298 (carboxypeptidase A2) | *Salmo salar* | Carboxypeptidase A2 precursor | 77.78% |
| comp192750_c0_seq2 | 1811 | 430 | K01298 (carboxypeptidase A2) | *Salmo salar* | Carboxypeptidase A2 precursor | 77.53% |
| comp171939_c0_seq1 | 1336 | 433 | K01300 (carboxypeptidase B2) | *Takifugu rubripes* | Carboxypeptidase B2-like | 71.18% |
| comp194055_c0_seq2 | 1567 | 450 | K01291 (carboxypeptidase B) | *Dicentrarchus labrax* | Carboxypeptidase B | 77.64% |
| comp190686_c0_seq1 | 4125 | 1048 | K01316 (protease, serine, 7 (enterokinase)) | *Danio rerio* | enteropeptidase | 54.24% |
| comp205417_c1_seq3 | 1731 | 494 | K01183 (chitinase) | *Danio rerio* | chitinase, acidic.3 precursor | 76.63% |
| comp205417_c1_seq1 | 1474 | 397 | K01183 (chitinase) | *Danio rerio* | chitinase, acidic.3 precursor | 76.52% |
| comp205417_c1_seq7 | 1821 | 476 | K01183 (chitinase) | *Danio rerio* | chitinase, acidic.3 precursor | 76.21% |
| comp205417_c1_seq5 | 1842 | 476 | K01183 (chitinase) | *Danio rerio* | chitinase, acidic.3 precursor | 76.84% |
| comp205417_c1_seq6 | 1862 | 476 | K01183 (chitinase) | *Lepisosteus oculatus* | acidic mammalian chitinase-like | 75.53% |
| comp205417_c1_seq4 | 1233 | 328 | K01183 (chitinase) | *Danio rerio* | Zgc:55406 protein | 72.78% |
| comp179586_c0_seq1 | 549 | 178 | K01183 (chitinase) | *Danio rerio* | Zgc:55406 protein | 61.36% |
| comp198374_c0_seq1 | 2094 | 455 | K01183 (chitinase) | *Lepisosteus oculatus* | acidic mammalian chitinase-like | 64.62% |
| comp198374_c0_seq3 | 2077 | 455 | K01183 (chitinase) | *Lepisosteus oculatus* | acidic mammalian chitinase-like | 64.62% |
| comp198374_c0_seq2 | 601 | 200 | K01183 (chitinase) | *Pundamilia nyererei* | acidic mammalian chitinase-like | 62.69 |
| comp148512_c1_seq1 | 395 | 131 | K01183 (chitinase) | *Drosophila mojavensis* | GI18891 | 43.41% |
| comp181770_c0_seq1 | 1690 | 552 | K01176 (alpha-amylase) | *Anguilla japonica* | alpha amylase | 99.61% |
| comp194127_c2_seq4 | 2041 | 446 | K01176 (alpha-amylase) | *Anguilla japonica* | alpha amylase | 74.82% |
| comp194127_c2_seq6 | 3367 | 515 | K01176 (alpha-amylase) | *Anguilla japonica* | alpha amylase | 74.9% |
| comp194127_c2_seq12 | 2073 | 515 | K01176 (alpha-amylase) | *Anguilla japonica* | alpha amylase | 74.9% |
| comp194127_c2_seq7 | 1177 | 296 | K01176 (alpha-amylase) | *Anguilla japonica* | alpha amylase | 74.23% |
| comp189307_c0_seq1 | 5769 | 1814 | K12047 (maltase-glucoamylase) | *Lepisosteus oculatus* | maltase-glucoamylase, intestinal-like | 73.23% |
| comp198159_c0_seq1 | 2022 | 471 | K14074 (pancreatic lipase-related protein 1) | *Anguilla japonica* | triglyceride lipase | 99.57% |
| comp175658_c0_seq1 | 787 | 116 | K14460 (colipase) | *Lepisosteus oculatus* | colipase-like | 71.93% |
| comp202779_c0_seq1 | 1935 | 561 | K12298 (bile salt-stimulated lipase) | *Thunnus orientalis* | bile salt-activated lipase 1 | 69.68% |
| comp198001_c0_seq1 | 2251 | 398 | K01052 (lysosomal acid lipase/cholesteryl ester hydrolase) | *Salmo salar* | Lysosomal acid lipase/cholesteryl ester hydrolase precursor | 78.09% |
| comp198001_c0_seq2 | 3354 | 398 | K01052 (lysosomal acid lipase/cholesteryl ester hydrolase) | *Salmo salar* | Lysosomal acid lipase/cholesteryl ester hydrolase precursor | 78.34% |
| comp198001_c0_seq5 | 3386 | 398 | K01052 (lysosomal acid lipase/cholesteryl ester hydrolase) | *Salmo salar* | Lysosomal acid lipase/cholesteryl ester hydrolase precursor | 78.09% |
| comp198001_c0_seq7 | 3369 | 398 | K01052 (lysosomal acid lipase/cholesteryl ester hydrolase) | *Salmo salar* | Lysosomal acid lipase/cholesteryl ester hydrolase precursor | 78.34% |
