# Supplementary material for: De Novo Assembly of the Whole Transcriptome of the Wild Embryo, Preleptocephalus, Leptocephalus, and Glass Eel of Anguilla japonica and Deciphering the Digestive and Absorptive Capacities during Early Development
Source: PLoS One. 2015 Sep 25;10(9):e0139105. doi: 10.1371/journal.pone.0139105 (PMC4583181; doi:10.1371/journal.pone.0139105)
Supplement: S3 Table — (DOCX) [file pone.0139105.s005.docx]

**S3 Table. Targeted transcripts of all digestive enzymes for expressional analysis and their FPKM values at different stages**

| Contig ID | Name of digestive enzyme | FPKM_preleptocephalus | FPKM_leptocephalus | FPKM_glass eel |
| --- | --- | --- | --- | --- |
| comp151497_c0_seq1 | pepsinogen (1) | 0.06 | 0.03 | 1.6 |
| comp167887_c0_seq1 | pepsinogen (2) | 0.04 | 0 | 0.79 |
| comp175835_c1_seq2 | trypsingen(1) | 0 | 0.71 | 0 |
| comp175835_c1_seq3 | trypsingen(2) | 0 | 1.11 | 0.19 |
| comp191757_c0_seq1 | chymotrypsin(1) | 3194.45 | 244.51 | 0.23 |
| comp195660_c0_seq7 | chymotrypsin(2) | 597.18 | 50.31 | 0.06 |
| comp177953_c0_seq1 | chymotrypsin-like elastase family member 3B-like | 149.76 | 8.98 | 0.49 |
| comp192750_c0_seq1 | carboxypeptidase A2(1) | 62.02 | 3.39 | 0.24 |
| comp192750_c0_seq2 | carboxypeptidase A2(2) | 87.04 | 4.04 | 0.16 |
| comp194055_c0_seq2 | carboxypeptidase B | 735.3 | 44.35 | 0.66 |
| comp190686_c0_seq1 | enteropeptidase | 10.85 | 1.7 | 0.53 |
| comp198374_c0_seq2 | chitinase, acidic.3 | 0 | 0 | 1.14 |
| comp181770_c0_seq1 | α-amylase | 6.78 | 2.55 | 0.25 |
| comp189307_c0_seq1 | maltase-glucoamylase, intestinal-like | 5.58 | 0.46 | 3.11 |
| comp198159_c0_seq1 | triglyceride lipase | 28.87 | 4.29 | 0.32 |
| comp175658_c0_seq1 | colipase | 20.98 | 20.77 | 0.08 |
| comp202779_c0_seq1 | bile salt-activated lipase 1 | 22.78 | 4.01 | 0.17 |
| comp198001_c0_seq7 | lysosomal acid lipase/cholesteryl ester hydrolase | 0 | 5.75 | 4.83 |
