# Supplementary material for: De Novo Assembly of the Whole Transcriptome of the Wild Embryo, Preleptocephalus, Leptocephalus, and Glass Eel of Anguilla japonica and Deciphering the Digestive and Absorptive Capacities during Early Development
Source: PLoS One. 2015 Sep 25;10(9):e0139105. doi: 10.1371/journal.pone.0139105 (PMC4583181; doi:10.1371/journal.pone.0139105)
Supplement: S4 Table — (DOCX) [file pone.0139105.s006.docx]

**S4 Table. Partial annotation of all targeted transcripts of nutrient transporters existing in the digestive tract**

| Contig ID | Contig length(bp) | Protein length (a.a.) | KEGG ID & name | (nr) Hit_organism | (nr) Hit_annotation | (nr) Identity |
| --- | --- | --- | --- | --- | --- | --- |
| comp206174_c2_seq2 | 4837 | 1163 | K14206 (solute carrier family 15 (oligopeptide transporter), member 1) | *Anguilla japonica* | peptide transporter 1 | 99.01% |
| comp204075_c0_seq2 | 3051 | 679 | K14210 (solute carrier family 3 (neutral and basic amino acid transporter), member 1) | *Salmo salar* | Neutral and basic amino acid transport protein rBAT | 70.97% |
| comp204543_c1_seq1 | 1786 | 100 | K13781 (solute carrier family 7 (L-type amino acid transporter), member 8) | *Danio rerio* | large neutral amino acids transporter small subunit 2 | 83.65% |
| comp204543_c1_seq6 | 779 | 259 | K13781 (solute carrier family 7 (L-type amino acid transporter), member 8) | *Xiphophorus maculatus* | large neutral amino acids transporter small subunit 2-like | 96.53% |
| comp193721_c0_seq1 | 1334 | 145 | K13781 (solute carrier family 7 (L-type amino acid transporter), member 8) | *Danio rerio* | large neutral amino acids transporter small subunit 2 | 84.46 |
| comp196520_c0_seq1 | 3589 | 698 | K14158 (solute carrier family 5 (sodium/glucose cotransporter), member 1) | *Salmo salar* | sodium/glucose cotransporter member 1 | 77.91% |
| comp192993_c0_seq1 | 1461 | 457 | K08143 (MFS transporter, SP family, solute carrier family 2 (facilitated glucose/fructose transporter), member 5) | *Oryzias latipes* | solute carrier family 2 (facilitated glucose/fructose transporter) member 5-like | 77.85% |
| comp192993_c0_seq2 | 1655 | 515 | K08143 (MFS transporter, SP family, solute carrier family 2 (facilitated glucose/fructose transporter), member 5) | *Oryzias latipes* | solute carrier family 2 (facilitated glucose/fructose transporter) member 5-like | 77.45% |
| comp190012_c2_seq1 | 399 | 132 | K07593 (MFS transporter, SP family, solute carrier family 2 (facilitated glucose transporter), member 2) | *Takifugu rubripes* | solute carrier family 2, facilitated glucose transporter member 2-like | 79.65% |
| comp190012_c2_seq2 | 2861 | 503 | K07593 (MFS transporter, SP family, solute carrier family 2 (facilitated glucose transporter), member 2) | *Dicentrarchus labrax* | solute carrier family 2 facilitated glucose transporter member 2 | 75.59% |
| comp206629_c0_seq2 | 3811 | 1090 | K14461 (Niemann-Pick C1-like protein 1) | *Xiphophorus maculatus* | niemann-Pick C1-like protein 1-like | 78.62% |
| comp206629_c0_seq1 | 4631 | 1090 | K14461 (Niemann-Pick C1-like protein 1) | *Xiphophorus maculatus* | niemann-Pick C1-like protein 1-like | 78.62% |
