# Supplementary material for: De Novo Assembly of the Whole Transcriptome of the Wild Embryo, Preleptocephalus, Leptocephalus, and Glass Eel of Anguilla japonica and Deciphering the Digestive and Absorptive Capacities during Early Development
Source: PLoS One. 2015 Sep 25;10(9):e0139105. doi: 10.1371/journal.pone.0139105 (PMC4583181; doi:10.1371/journal.pone.0139105)
Supplement: S5 Table — (DOCX) [file pone.0139105.s007.docx]

**S5 Table. Targeted transcripts of each nutrient transporter for expressional analysis and its FPKM value at different stages**

| Contig ID | Name of nutrient transporter | FPKM_preleptocephalus | FPKM_leptocephalus | FPKM_glass eel |
| --- | --- | --- | --- | --- |
| comp206174_c2_seq2 | peptide transporter 1 | 50.78 | 2.63 | 2.7 |
| comp204075_c0_seq2 | neutral and basic amino acid transport protein rBAT | 13.09 | 5.09 | 0.21 |
| comp204543_c1_seq1 | large neutral amino acids transporter small subunit 2 | 12.42 | 3.68 | 37.08 |
| comp196520_c0_seq1 | sodium/glucose cotransporter member 1 | 3.51 | 4.27 | 1.49 |
| comp192993_c0_seq1 | solute carrier family 2 (facilitated glucose/fructose transporter) member 5-like (1) | 0.8 | 0 | 0.37 |
| comp192993_c0_seq2 | solute carrier family 2 (facilitated glucose/fructose transporter) member 5-like (2) | 1.95 | 0.25 | 0.42 |
| comp190012_c2_seq2 | solute carrier family 2 facilitated glucose transporter member 2 | 2.09 | 3.14 | 2.31 |
| comp206629_c0_seq1 | niemann-Pick C1-Like protein 1 (1) | 5.63 | 2.58 | 0.19 |
| comp206629_c0_seq2 | niemann-Pick C1-Like protein 1 (2) | 2.09 | 0.03 | 0 |
